# Spin angular momentum modulation via spin–orbit interaction in fractional orbital angular momentum beams

**DOI:** 10.1515/nanoph-2025-0430

**Published:** 2025-11-10

**Authors:** Xusheng Chen, Fanfei Meng, Kang Du, Min Lin, Luping Du

**Affiliations:** Nanophotonics Research Centre, Institute of Microscale Optoelectronics, Shenzhen University, Shenzhen, 518060, China; School of Physical Sciences, 636498 Great Bay University, Dongguan, 523000, China; College of Integrated Circuits and Optoelectronic Chips, Shenzhen Technology University, Shenzhen, 518118, China

**Keywords:** spin angular momentum, spin–orbital interaction, fractional orbital angular momentum

## Abstract

Light exhibits both spin and orbital angular momentum (SAM and OAM). These two forms of angular momentum remain independent in paraxial fields, but become coupled in confined fields through spin–orbit interactions (SOI). The SOI mechanism allows for the manipulation of SAM to generate structured light fields featuring nontrivial topological characteristics, such as optical skyrmions. Conventional OAM beams, nonetheless, carry discrete integer topological charges (TCs), leading to discrete SAM states. This discrete property poses a persistent challenge for achieving continuous control of SAM. To tackle this fundamental issue, we explored fractional orbital angular momentum (FOAM) beams, whose TCs are extended from integers to fractions, to realize continuous and precise control of SAM. A direct mathematical relationship between the fractional effective TCs of FOAM beams and the orientation distributions of the SAM vector has been derived. This theoretical prediction has been experimentally verified using our home-built near-field mapping system, by which the distinct SAM distributions of surface cosine waves regulated by FOAM beams were mapped out. As a potential application, we also devised an inverse detection method to accurately measure the fractional effective TCs of FOAM, which achieved theoretical and experimental accuracies of 10^−5^ and 10^−2^, respectively. These advancements may enhance our fundamental understanding of the SOI mechanism, and hence could create novel opportunities for light field manipulation, optical communication, and other related areas.

## 1 Introduction

Since the groundbreaking work by J. H. Poynting, light has been recognized as carrying momentum and angular momentum [1]. Subsequently, the discovery of orbital angular momentum (OAM) in photon wavefunctions has established a new paradigm in modern photonics [2], [3], [4], [5], [6], [7]. Beams who carry OAM are characterized by their helical phase fronts *e* ^*ilϕ*^, where *l* represents the discrete topological charge (TC), and each photon is endowed with *lℏ* (where ℏ is the reduced Planck’s constant) of OAM [8]. Beyond OAM, light inherently possesses spin angular momentum (SAM), which is fundamentally associated with the helicity of polarization [1], [9], [10], [11], [12]. SAM is typically manifested through circular polarizations. Specifically, right-handed and left-handed circularly polarized (RCP and LCP) light carry +ℏ and −ℏ of SAM per photon, respectively [13]. Generally, OAM and SAM are independent in paraxial conditions. However, this independent conservation can be violated in tightly focused beams [14], [15], evanescent waves [12] and scattering configurations [16]. In these cases, SAM and OAM of photons are no longer conserved separately. Instead, they can undergo mutual conversion through spin–orbit interactions (SOI) [17], [18], [19], [20]. Notably, this SOI mechanism enables sophisticated manipulation of the SAM characteristics. For instance, the manipulation of transverse spin [21], [22] can generate intricate photonic spin textures, including skyrmions [23], [24], [25], [26], merons [27], [28], [29], hopfions [30], torons [31], as well as other topologically nontrivial structures [32], [33].

Although SOI allows for the manipulation of SAM through the modulation of OAM, the discrete property of angular momentum intrinsically restricts SAM to discrete states [23], [29], [34]. As a result, attaining continuous and arbitrary control over the SAM vector remains a fundamental challenge in the field of photonics. Recent advances in OAM have transcended the integer limitations through fractional effective TCs, defining fractional orbital angular momentum (FOAM) beams as structured light fields that carry OAM with fractional effective TCs [35], [36], [37], [38], [39]. The fractional vortex phase term is typically expressed as a superposition of integer vortex phase basis states [40]. Therefore, FOAM beams do not violate the quantum nature of angular momentum, but rather achieve phase modulation through superposition of multiple angular momentum states. Significantly, this configuration simultaneously creates opportunities for continuous manipulation of the SAM vectors.

In this paper, we demonstrate the continuous manipulation of spin textures within surface Cosine beams by making use of FOAM beams. By extending TCs *l* from discrete integers to continuous fractions, smooth and arbitrarily precise control of SAM distribution is achieved. Theoretically, we have established a direct mathematical connection between the fractional effective TCs of FOAM beams and SAM vector orientation distributions of surface waves. This fundamental relationship provides the theoretical basis for achieving continuous SAM modulation via FOAM beams. Meanwhile, experimental validation was accomplished through precise measurements of SAM distributions by our home-built near-field scanning system. Finally, an approach for accurate OAM detection through SAM measurements in SOI systems was developed, with theoretical identification accuracy reaching 10^−5^ and experimental realization attaining 10^−2^. The proposed method offers new opportunities for the development of advanced optical systems, especially in the fields of light field manipulation, optical communications, and beyond.

## 2 Results and discussions

**Figure 1:**
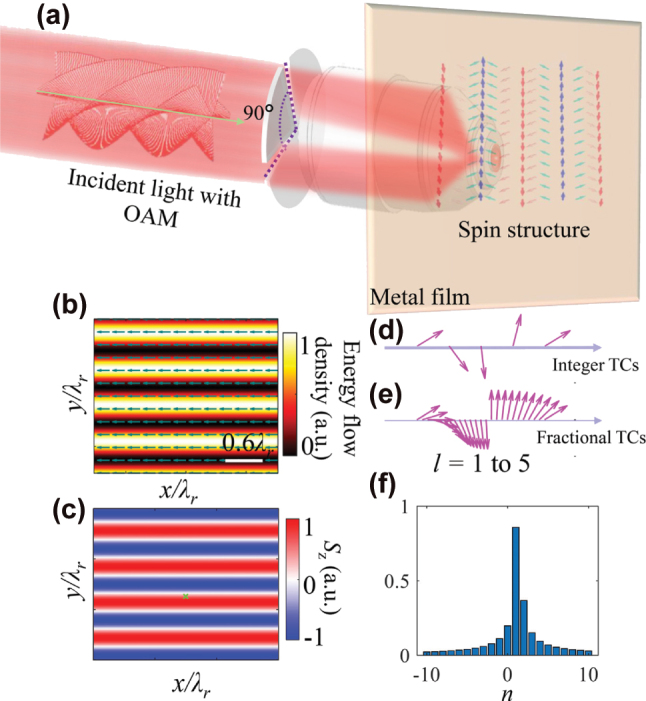
Continuous modulation of SAM by fractional OAM beam. (a) Schematic diagram of the proposed setup for generating the surface cosine beam. The intensity mask divides an OAM beam into two parts propagating by an azimuthal angle of 90°. When focused onto the metal film, their interference generates cosine-type surface waves. Through spin–orbit interaction, these surface waves subsequently induce periodic spin textures. (b) The spatial distributions of the energy flow density for the surface cosine beam. The arrows indicate the direction of energy flow. (c) The distribution of the out-of-plane SAM components (*S*_z_). (d–e) The variations of the SAM vector at a given point [marked by green cross in (c)] evolves as the topological charge varies from *l* = 1 to 5 (*x*-axis), when TCs are integers (d) or fractions (e), respectively. (f) Coherence phase spectrum by coherence phase is decomposed.

Suppose a vortex beam with radial polarization is blocked by an intensity mask, and is divided into two parts by the two slits of the mask, as depicted in Figure 1(a). The azimuthal angle between these two slits is set at 90° (π/2). Following the tight focusing process using a high numerical aperture (NA) objective lens, two surface plasmon polariton waves with optical vortices (SPP-OVs) propagating at perpendicular propagation directions are generated at the metal–dielectric interface. Given that the shape of the interference fringes is a cosine stripe, we term it a cosine beam.

This process can be mathematically described by an interferometric Hertz vector potential formulation in a source-free, homogeneous medium as [41]

(1) $$\begin{array}{ll} \Psi & =\Psi_{1}+\Psi_{2}=A_{1}e^{il\varphi_{1}}{\text{e}}^{\text{i}k_{r}r\cdot e_{1}}e^{-k_{z}z}\\ & \;+A_{2}e^{il\varphi_{2}}{\text{e}}^{\text{i}k_{r}r\cdot e_{2}}e^{-k_{z}z} \end{array}$$

where *A*_1_ = *A*_2_ = 1, *φ*_1_ = 3*π*/4, *φ*_2_ = 5*π*/4, $e_{1}=\left(\cos\varphi_{1},\sin\varphi_{1}\right)$ , $e_{2}=\left(\cos\varphi_{2},\sin\varphi_{2}\right)$ , $r=\left(x,y\right)$ is in-plane coordinate. Symbols *k*_*r*_ and *ik*_*z*_ denote the transverse and longitudinal wavevector components, respectively, and satisfy $k_{r}^{2}-k_{z}^{2}=k^{2}$ with *k* denoting the free-space wave vector. The symbol *l* is the topological charge of the SPP-OV. The time averaging Poynting vector $P=\text{Re}\;\left(E^{*}\times H\right)/2$ representing a directional energy flux can be calculated through the Hertz potential as [28]:

(2) $$P=\frac{\omega \varepsilon k_{r}^{2}}{2}\text{Im}\;\left(\Psi^{*}\nabla \Psi \right)$$

where *ω* is the angular frequency of the wave and *ɛ* is the absolute permittivity of the medium. The generalized spin-momentum relation between the Poynting vector and SAM gives rise to [21], [42]:

(3) $$\begin{array}{ll} S & =\frac{1}{2\omega^{2}}\nabla \times P=\frac{\varepsilon k_{r}^{2}}{4\omega }\text{Im}\;\left(\nabla \Psi^{*}\times \nabla \Psi \right)\\ & =\frac{\varepsilon k_{r}^{3}e^{-2k_{z}z}}{2\omega }\left(\begin{array}{c} 0\\ \sqrt{2}k_{z}\left(1+\cos\left(\frac{l\pi }{2}-\sqrt{2}k_{r}y\right)\right)\\ k_{r}\sin\left(\frac{l\pi }{2}-\sqrt{2}k_{r}y\right) \end{array}\right) \end{array}$$

where the TC *l* can be either an integer or a fraction.

The energy flow distribution calculated by Eq. (2) is presented in Figure 1(b). This distribution determines the transverse SAM via Eq. (3), thereby constructing a three-dimensional spin vector distribution illustrated on the right side of Figure 1(a). For integer TCs (*l* = 1, 2, 3, 4…), the SAM vector cyclically experiences four discrete orientations and resets when *l* = 5, indicating a modulo-4 periodicity. This periodic law stems from the fact the propagation direction between the two SPP wave is 90° [also determines $\frac{l\pi }{2}$ in Eq. (3)], which are analogous to the determination law of four-fold symmetry for square spin meron lattice [28], [29]. In addition, this angle also determines that the spatial frequency of SAM distribution is $\sqrt{2}k_{r}$ at the same time. To visualize this behavior, Figure 1(c) presents the *S*_z_ distribution of the SPP-OV for *l* = 1, while Figure 1(d) tracks the spin-vector evolution at a fixed point [marked by green cross in Figure 1(c)] when *l* ranges from 1 to 5.

Furthermore, if the TC *l* is extended from integer to fraction, that is, when the incident light is FOAM beam, Eq. (3) still holds. In this case, continuously varying SAM vectors states can be generated between the discrete states that result from integer TCs. It should be mentioned that FOAM do not violate the quantum nature of angular momentum and can be understood by a superposition of multiple integer OAM modes, with their phase profile described by [36]:

(4) $${\text{e}}^{il\phi }=\frac{{\text{e}}^{\text{i}\pi l}\sin\left(\pi l\right)}{\pi }\overset{\infty }{\underset{n=-\infty }{\sum }}\frac{e^{in\phi }}{l-n}$$

where *n* is an integer that denotes the topological charge in the OAM spectrum. The coherent superposition of multiple TC states as expressed in Eq. (4) produces smooth phase gradients, generating FOAM modes with continuously variable “effective” TC values [e.g., *l* = 1.3 in Figure 1(f)]. This continuous phase modulation fundamentally modifies the transverse Poynting vector distribution, enabling smooth rotation of the SAM vector [Figure 1(e)] instead of the discrete transitions characteristic as shown in Figure 1(d). Through this mechanism, the FOAM achieves analog control of SAM orientation (similar to the continuous movement of an analog clock as opposed to the discrete steps of a digital clock) by establishing continuously tunable SOI. This approach provides precise control of photonic spin states that surpasses the fundamental limitations of integer OAM systems.

**Figure 2:**
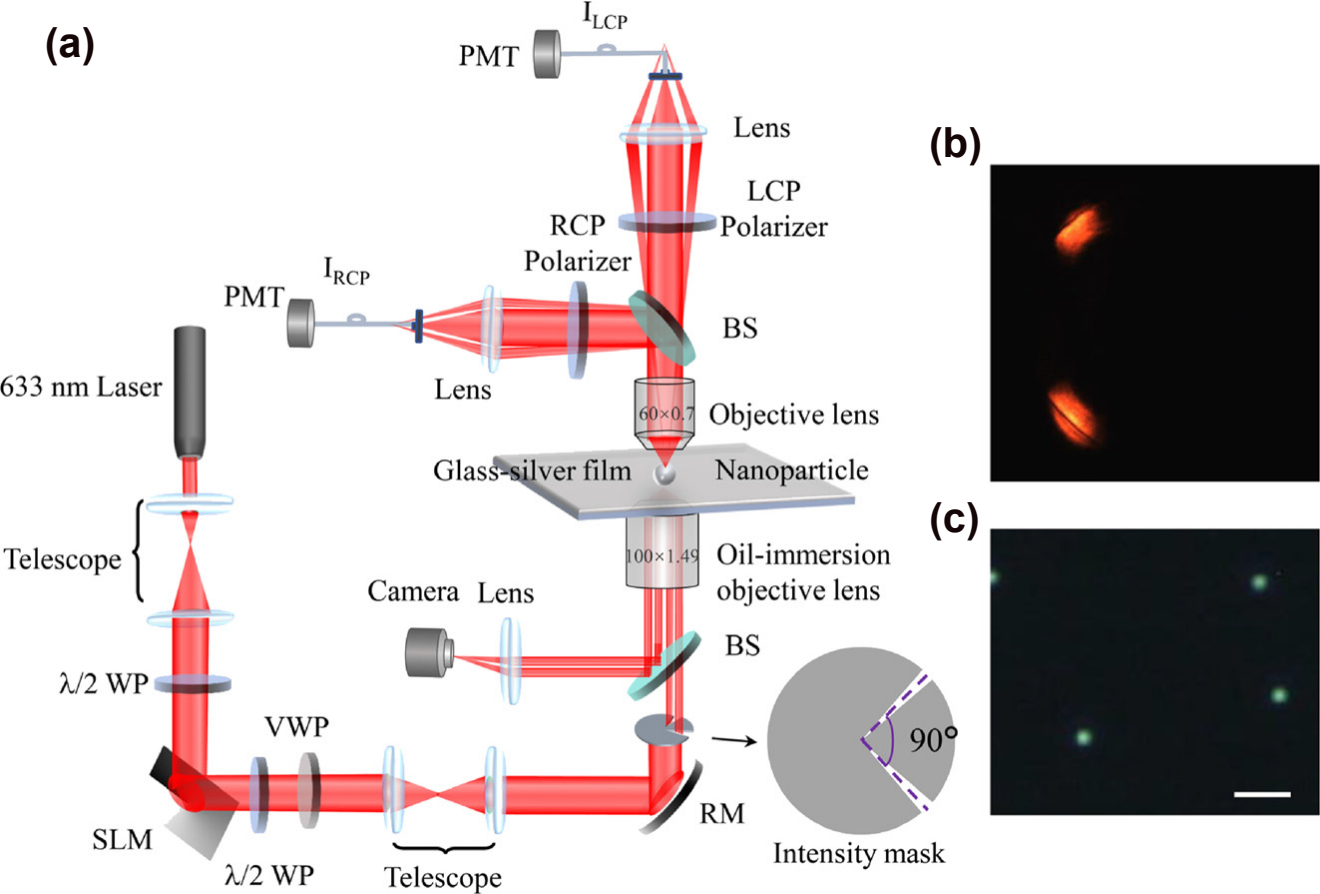
Key elements for the experiments. (a) The diagram of the experimental setup for characterizing the spin distribution in a surface plasmon vortex. (b) Back focal plane image of the reflected beam from the oil-immersion objective lens, where the dark-line indicates the excitation of SPPs at the air-gold interface. (c) Dark field image of the isolated PS nanospheres immobilized on the gold film. The scale bar represents 1 μm. SLM: Spatial light modulator, *λ*/2 WP: Half-wave plate, VWP: vortex wave plate, RM: reflect mirror, BS: beam splitter (non-polarizing), PMT: photo-multiplier tube.

A schematic diagram of the experimental setup employed to demonstrate the spin distribution is presented in Figure 2(a). The setup commences with a 633 nm laser source, followed by a telescope system and a half-wave plate. The beam then shines on a spatial light modulator (SLM, UPO: HDSLM80R Plus) and reflects a beam with phase vortices. After using another half-wave plate and a vortex wave plate to generate a radially polarized vortex beam with optical phase vortices (RPOV), the beam then encounters an intensity mask (angular aperture less than 10°) and thus splits into two parts with a relative angle of 90°. Finally, the beam is tightly focused by an oil-immersion objective lens (Olympus, 100×, NA = 1.49) onto the SPP waveguide, which is a 50 nm-thick gold film on a silica substrate. This process excites SPP waves at the air-gold interface, and the image captured from the back focal plane is shown in Figure 2(b), where the dark lines indicate the excitation of SPP waves at the interface. A polystyrene (PS) nanoparticle of a diameter of ∼320 nm is immobilized onto the gold surface to scatter the SPPs into far field for detection. The image of the nanoparticle in the dark-field mode of the system is shown in Figure 2(c). The scattered light carrying the local spin information is collected by another objective lens (Olympus, 50×, NA = 0.7), and is split into two arms, each equipped an LCP and an RCP polarizer, respectively, to selectively filter out LCP and RCP light. Ultimately, the intensity of the LCP and RCP components is collected by photo-multipliers (PMT). Note that both the dipole moment of the 320 nm PS nanoparticle and its directional radiation, along with the collection objective lens, results in the precise near field measurement, which our previous work discussed in detail [43].

**Figure 3:**
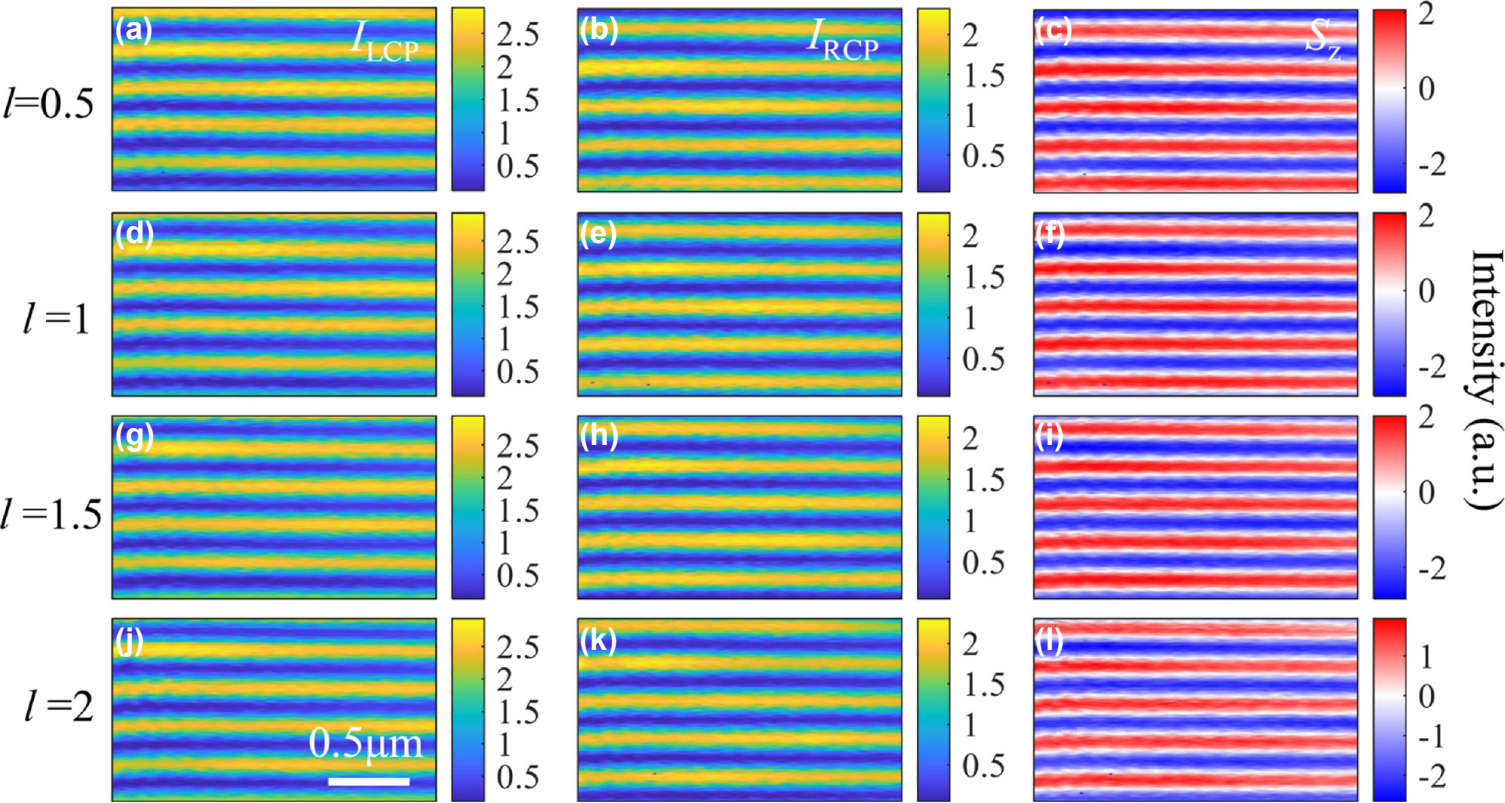
The experimental results for the SPPs that are excited by RPOV beam with (a–c) 0.5-order vortex phase, (d–f) 1-order vortex phase, (g–i) 1.5-order vortex phase, and (j–l) 2-order vortex phase, respectively. The left and middle panels show the measured intensity of LCP and RCP component of SPPs, and the right panel shows the resultant *z*-component of SAM (*S*_z_).

The SAM of an arbitrary SPP wave can be calculated by $S=\left\{\varepsilon \left.E^{*}\times E+\mu H^{*}\times H\right\}\right./4\omega$ , where the asterisk (*) stands for the complex conjugate, and *μ* is permeability of the medium. Consequently, the *z*-component of SAM is related to the in-plane circularly polarized components described in [44]:

(5) $$S_{z}=\frac{\varepsilon }{4\omega i}\frac{k_{r}^{2}}{k_{z}^{2}}\left(E_{x}^{*}E_{y}-E_{y}^{*}E_{x}\right)=\frac{\varepsilon }{4\omega }\frac{k_{r}^{2}}{k_{z}^{2}}\left(I_{\text{RCP}}-I_{\text{LCP}}\right)$$

where *I*_RCP_ and *I*_LCP_ indicates the intensity of RCP and LCP components, respectively. In a word, the out-of-plane SAM (*S*_z_) of SPP field can be uncovered by simply measuring the intensity of the LCP and RCP components obtained from the in-plane electric field. Figure 3(a) and (b) depict the measured LCP and RCP intensity distribution, when *l* = 0.5. Figure 3(c) shows the *S*_z_ distribution which is proportional to the subtraction of the former two distributions, as per Eq. (4). Meanwhile, Figure 3 presents the *S*_z_ distributions when incident beam carries FOAM with variable TCs, ranging from *l* = 0.5 to 2 (from top to bottom). Evidently, by varying the frctional TCs, the displacement of the cosine-shaped *S*_z_ distribution can be continuously adjusted. In another perspective, a fixed point experiences a continuous evolution of the spin vector, as well. These results are consistent and have validated the theoretical predictions put forward above.

**Figure 4:**
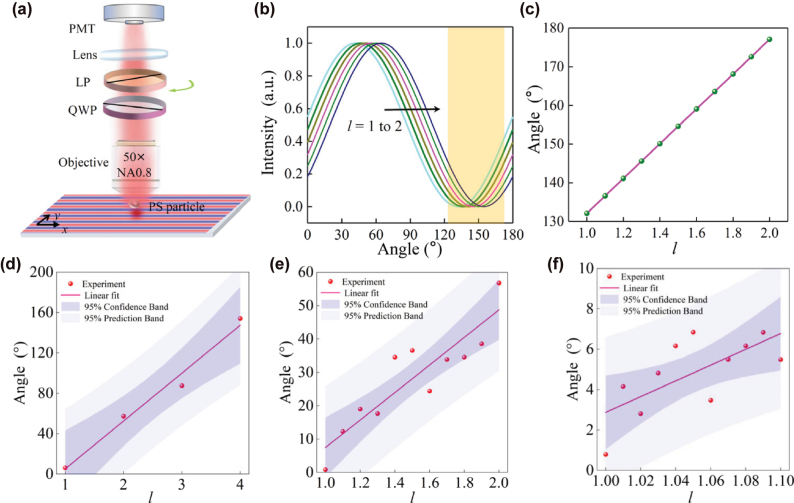
A proof-of-concept experiment for detecting discrimination of OAM modes. (a) Schematic of the setup showing the collection part, while the excitation part is shown in Figure 2(a). QWP: quarter wave plate, LP: linear polarizer, PMT: photo-multiplier tube. (b) The polarizer-rotation-dependent intensity profile at a given point in the SPP field demonstrates an angular displacement as the TC of the OAM transitions from 1 to 2. (c) Theorical relationship between the polarizer orientation angles for intensity minima and the fractional effective TCs of FOAM, who lie in the yellow-shaded region in (b). (d–f) Measured angular values as a function of TC *l*, ranging from 1 to 4 with (d) 1-step increment, (e) 0.1-step increment, and (f) 0.01-step increment, respectively. Experimental data with linear regression fit, along with 95 % confidence and prediction bands are also presented.

Finally, as a potential application of our theory, we put forward an approach for accurately discriminating OAM modes in vortex beams via SAM measurements in the SOI system. The configuration is derived from the setup described in Figure 2. Nevertheless, in the collection part, the scattered radiation from the PS nanoparticle was collected using an objective lens (Olympus, 50×, NA = 0.8). Subsequently, it was analyzed through a polarization measurement system composed of a fixed quarter-wave plate and a linear polarizer mounted on a rotation stage (Thorlabs: DDR25) to measure the elliptical polarization state of the scattered light [Figure 4(a)]. A characteristic intensity modulation curve is observed when rotating the linear polarizer [Figure 4(b)], exhibiting a distinct intensity dip (e.g., in the highlighted yellow region). The angle corresponding to the dip increases with the TC value increases. Figure 4(c) illustrates this theoretical linear relationship between the angular position of the intensity minimum and the TC value. For each increment of 1 in the TC, a polarization rotation of 45° is induced. Consequently, the high-precision rotation stage (minimum incremental rotation of 0.00036°) yields a theoretical resolution limit of Δ*l*_theo_ ≈ 1 × 10^−5^ for TC detection. In the experiment, we carried out comprehensive measurements of TC variations. Initially, we increased TC in integer steps from 1 to 4 [Figure 4(d)]. Subsequently, we performed scans with a finer 0.1-step interval between *l* = 1 to 2 [Figure 4(e)]. Finally, we achieved high-precision characterization through 0.01-step measurements within the TC range of 1–1.1 [Figure 4(f)]. This TC detection precision of Δ*l*_exp_ ≈ 0.01 was essentially constrained by the phase modulation precision of the employed SLM. In Figure 4(d–f), the red dots represent the measured data, while the red lines denote the fitting curves with 95 % confidence bands and prediction bands. The slopes of these fitted lines are all approximately 45° per TC, demonstrating good agreement with theoretical predictions. Notably, there are relatively large errors in some of the data in Figure 4(f). These errors mainly stem from system vibrations, which hindered the stable fixation of the measurement position. Nevertheless, considering that the theoretical TC detection precision can reach 1 × 10^−5^, we believe that this inverse method based on SAM measurement has the potential to outperform the conventional OAM spectral characterization methods [40], [45], [46], [47] with the system stability and the generated phase modulation resolution are improved.

## 3 Conclusions

In conclusion, we have established a fundamental framework for continuous manipulation of the SAM by leveraging the unique properties of FOAM beams. As a proof-of-concept, we investigated surface cosine beams to demonstrate the proposed spin-momentum locking effect. This effect creates a deterministic relationship between the fractional effective TCs of the FOAM and the vector characteristics of the SAM. By using FOAM beams, we achieved smooth and arbitrarily precise manipulation of the orientation of the SAM, thereby overcoming the discrete constraint inherent in conventional integer OAM beams. Subsequently, we experimentally validated our findings through precise measurements of the SAM distributions using our home-built near-field scanning system. Furthermore, we develop a derived metrological approach for FOAM detection via SAM measurements in the SOI system. Due to the ultrasensitive nature of the SOI occurs in near fields, theoretical and experimental resolutions of 10^−5^ and 10^−2^ were achieved, respectively. These advancements may enhance our comprehension of the angular momentum of photonics and may also unlock transformative applications in optical manipulation, quantum state encoding, and topological photonics, where FOAM beams enable arbitrary spin control and complex singularity engineering.
